# Supplementary material for: Ganoderma lucidum Immobilized on Wood Demonstrates High Persistence During the Removal of OPFRs in a Trickle-Bed Bioreactor
Source: J Fungi (Basel). 2025 Jan 22;11(2):85. doi: 10.3390/jof11020085 (PMC11856180; doi:10.3390/jof11020085)
Supplement: Supplementary file 1 [file jof-11-00085-s001.zip › jof-3334684-supplementary.pdf]

Supplementary Material

Ganoderma lucidum Immobilized on Wood Demonstrates High Persistence During the Removal of OPFRs in a Trickle-Bed Bioreactor

Shamim Tayar[1], Javier Villagra[2], Núria Gaju[2], Maira Martínez-Alonso[2], Eduardo Beltrán-Flores[1] and Montserrat Sarrà[1*]

[1] Departament d'Enginyeria Química Biològica i Ambiental, Escola d'Enginyeria, Universitat Autònoma de Barcelona, 08193 Bellaterra, (Barcelona), Spain. (Shamim.Tayar@uab.cat), (Eduardo.Beltran@uab.cat)

[2] Departament de Genètica i Microbiologia, Universitat Autònoma de Barcelona, 08193 Bellaterra, (Barcelona), Spain (Javier.Villagra@uab.cat) (Nuria.Gaju@uab.cat) (Maira.Martinez@uab.cat)

*Corresponding Author.

E-mail address: Montserrat.Sarra@uab.cat

Phone number: +345812789

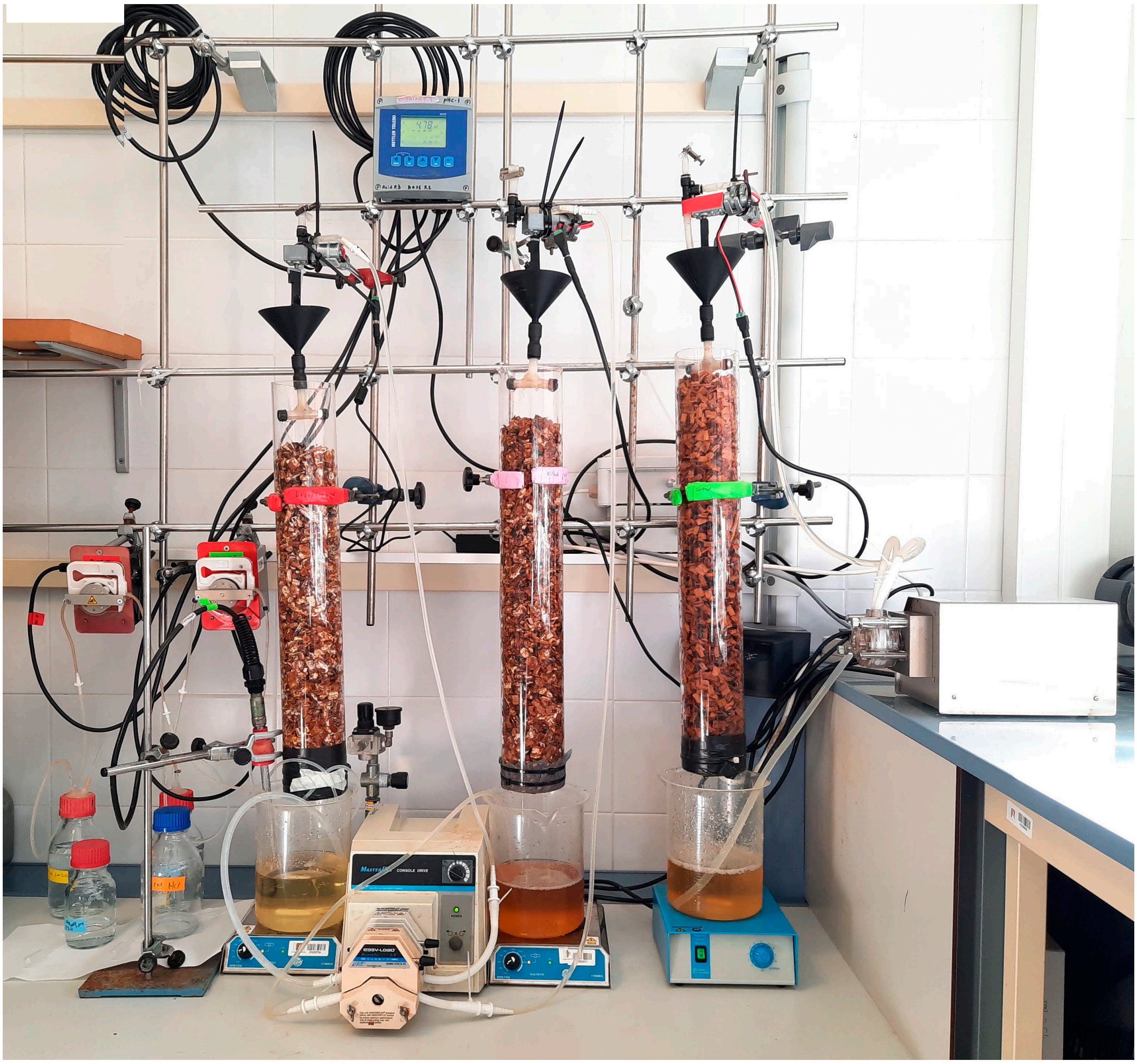

Figure S1. The lab-scale trickle bed reactor (TBR).

Table S1. PCR and qPCR conditions

Primer pair	Target group	Primer sequences 5'-3'	Cycling conditions	References[c]
EF4<br>ITS4	Fungal ITS region	GGAAGGGRTGTATTTATTAG<br>TCCTCCGCTTATTGATATGC	Initial denaturation: 94ºC 5 min; 94ºC 30s, 55ºC 30s, 72ºC 30s (35 cycles) and final elongation 72ºC 5 min	1
GC-ITS1F<br>ITS2R	Fungal ITS region	GC clamp[a] TTGGTCATTTAGAGGAAGTAA<br>GCTGCGTTCTTCATCGATGC	Initial denaturation: 94ºC 5 min; 94ºC 30s, 55ºC 30s, 72ºC 30s (35 cycles) and final elongation 72ºC 5 min	2
GC-341F<br>907RM	Bacterial 16S rDNA	GC clamp[b] CCACGGGAGGCAGCAG<br>CCGTCAATTCMTTTGAGTTT	Initial denaturation: 94ºC 5min; 94ºC 1 min, 65ºC 1min [-0,5ºC each cycle], 72ºC 3min (20 cycles); 94ºC 1 min, 55ºC 1min, 72ºC 3 min (15 cycles) and final elongation 72ºC 7 min	3
ITS3<br>ITS4	Fungal ITS region	GCATCGATGAAGAACGCAGC<br>TCCTCCGCTTATTGATATGC	Initial denaturation: 95ºC 1 min; 94ºC 30s, 54ºC 40s, 72ºC 40s (40 cycles)	4
Com1<br>769R	Bacterial 16S rDNA	CAGCAGCCGCGGTAATAC<br>ATCCTGTTTGMTMCCCVCRC	Initial denaturation: 95ºC 10 min; 95ºC 10s, 59ºC 10s, 72ºC 12s (40 cycles)	5
ITS1F<br>Gano2R	*Ganoderma sp.* ITS region	CTTGGTCATTTAGAGGAAGTAA<br>TATAGAGTTTGTGATAAACGCA	Initial denaturation: 95ºC 5 min; 95ºC 45s, 60ºC 45s [-0.5ºC each cycle], 72ºC 45s (10 cycles); 95ºC 45s, 55ºC 45s, 72ºC 45s (25 cycles)	6

Note: a: GC clamp: CGCCGCCGCGCGCGGCGGGCGGGGCGGGGGCACGGGGGGC.
b: GC clamp: CGCCCGCCGCGCCCCGCGCCCGGCCCGCCGCCCCCGCCCC.
c:1 (Gardes et al., 1993); 2 (Withe et al., 1990); 3 (Muyzer et al., 1996, 1993); 4 (Maza-Márquez et al., 2018); 5 (Dorn-In et al., 2015) and 6 (modified from Guglielmo et al., 2007).

Table S2. TCEP residual concentrations and laccase activities detected in the liquid phase.

G. lucidum				*T. versicolor*			
Time (days)	TCEP liquid (mg/L)	Removal (%)	Laccase activity (AU/L)	Time (days)	TCEP liquid (mg/L)	Removal (%)	Laccase activity (AU/L)
0	9.79	0	0	0	9.79	0	0
7	7.17±0.28	26.76	4.73	7	6.88±0.51	29.72	0

Table S3. Chromatographic characteristics of the TPs of TBP by *T. versicolor* and *G. lucidum* in Erlenmeyer flasks after 7d incubation

Identified TP	Monoisotopic mass	Retention time (min)	Measured mass (m/z)	Mass error (ppm)	Molecular formula	RDB
Dibutyl 3-hydroxybutyl phosphate (OH-TBP)	282.1596	7.71	283.1667	– 0.590	$C_{12}H_{28}O_5P$	– 0.5
			265.1562	– 0.386	$C_{12}H_{26}O_4P$	0.5
			211.1091	– 1.336	$C_8H_{20}O_4P$	– 0.5
			153.0310	– 0.535	$C_4H_{10}O_4P$	0.5
			98.9841	– 0.825	H_4O_4P	– 0.5
Dibutyl phosphate (DBP)	210.1021	4.80	211.1091	– 1.194	$C_8H_{20}O_4P$	– 0.5
			155.0466	– 1.302	$C_4H_{12}O_4P$	– 0.5
			98.9840	– 1.330	H_4O_4P	– 0.5
			80.9735	– 0.827	H_2O_3P	0.5
			57.0701	3.735	C_4H_9	0.5
Butyl dihydrogen phosphate (MBP)	154.0395	4.80	155.0465	– 1.689	$C_4H_{12}O_4P$	– 0.5
			98.9840	– 1.734	H_4O_4P	– 0.5
			80.9735	– 1.691	H_2O_3P	0.5
Butyl 3-hydroxybutyl phosphate (OH-DBP)	226.0970	2.81	227.1040	– 0.998	$C_8H_{20}O_5P$	– 0.5
			171.0414	– 1.616	$C_4H_{12}O_5P$	– 0.5
			155.0465	– 1.495	$C_4H_{12}O_4P$	– 0.5
			153.0310	– 0.861	$C_4H_{10}O_4P$	0.5
			98.9840	– 1.330	H_4O_4P	– 0.5
			73.0648	– 0.020	C_4H_9O	0.5
			55.0545	4.781	C_4H_7	1.5

Note: Molecular formulas were calculated on the basis of their accurate mass measurements and the observed isotopic patterns. Unsaturation degree was expressed as double bound equivalents (RDB).

Table S4. Chromatographic characteristics of the TPs of TCEP by *T. versicolor* and *G. lucidum*

Identified TP	Monoisotopic mass	Retention time (min)	Measured mass (m/z)	Mass error (ppm)	Molecular formula	RDB
Bis(2-chloroethyl) 2-hydroxyethyl phosphate (BCEP-OH)	265.99	5.37	266.9950	-0.981	$C_6H_{14}O_5Cl_2P$	-0.5
			222.9686	0.057	$C_4H_{10}O_4Cl_2P$	-0.5
			160.9763	-0.183	$C_2H_7O_4ClP$	-0.5
			124.9997	-0.253	$C_2H_6O_4P$	0.5
			98.9840	-0.218	H_4O_4P	-0.5
			62.9997	3.104	C_2H_4Cl	0.5
6-Chloro-5-oxohexyl dihydrogen phosphate	230.01	4.92	231.0181	-1.273	$C_6H_{13}O_5ClP$	0.5
			169.0258	-0.689	$C_4H_{10}O_5P$	0.5
			143.0102	-1.163	$C_2H_8O_5P$	-0.5
			124.9997	-1.053	$C_2H_6O_4P$	0.5
			98.9840	-1.633	H_4O_4P	-0.5
			95.0491	-0.857	C_6H_7O	3.5
			62.9997	2.152	C_2H_4Cl	0.5

Note: Molecular formulas were calculated on the basis of their accurate mass measurements and the observed isotopic patterns. The unsaturation degree was expressed as double-bound equivalents (RDB).

Table S5. COD and turbidity at the end of each 3d batch treatment in the TBR.

Batch	COD (mg/L)			A_{650}		
	Experimental treatment	Killed control	Abiotic control	Experimental treatment	Killed control	Abiotic control
1	5264	6755	5102	0,073	0,089	0,205
2	2715	3367	3241	0,053	0,065	0,146
3	1858	2313	2779	0,046	0,06	0,114
4	1548	1840	2448	0,04	0,041	0,083
5	1258	1470	1990	0,031	0,036	0,041
6	1344	1126	1756	0,029	0,036	0,034
7	1082	1027	1530	0,05	0,038	0,046
8	946	818	1376	0,021	0,03	0,038
9	938	806	1345	0,02	0,023	0,026
10	1011	938	1382	0,02	0,02	0,018

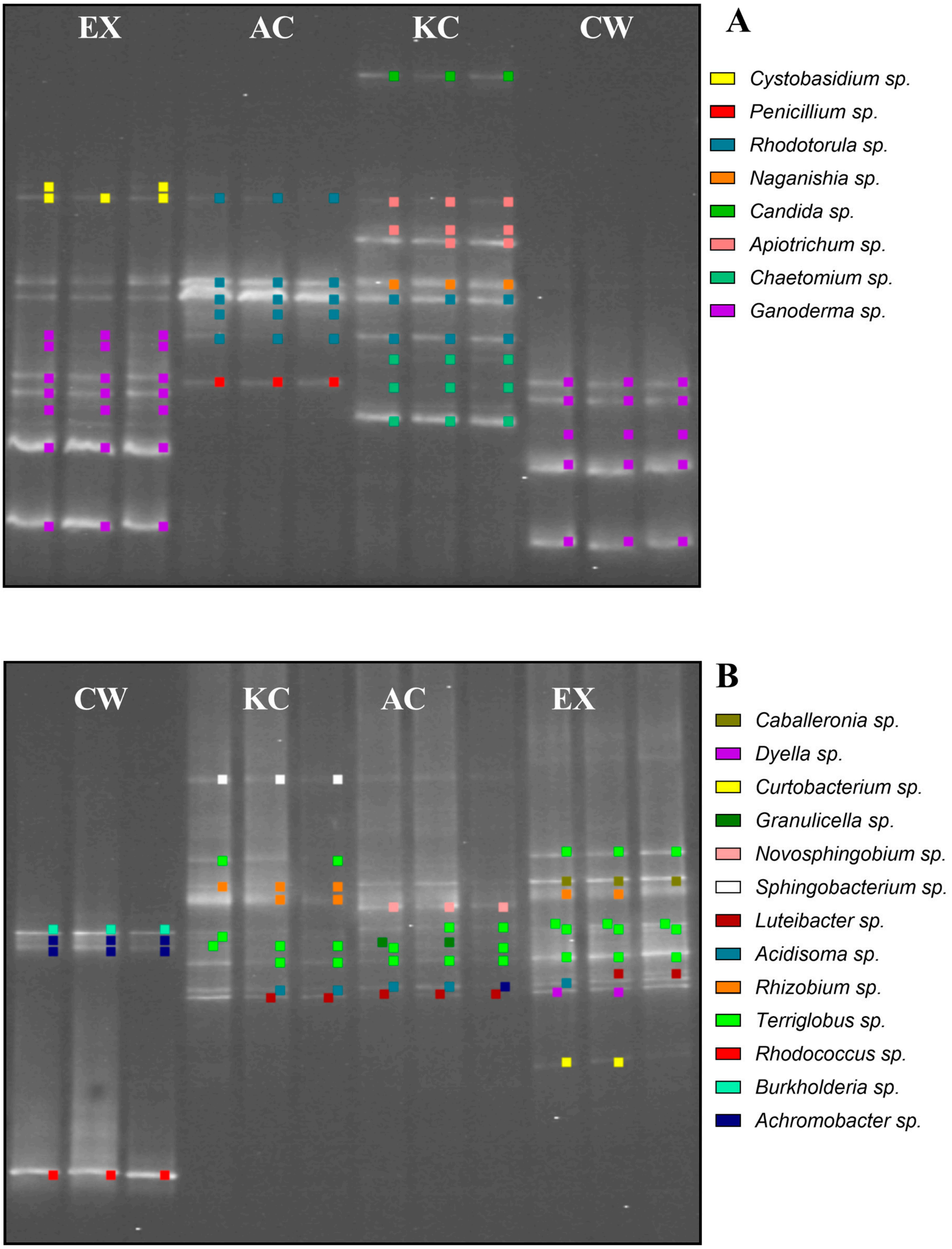

Figure S2. DGGE profiles of the fungal (A) and bacterial (B) communities found in the different treatments, each square mark a band that was recovered, sequenced, and uploaded to NCBI, colour indicates the taxonomical group at genus level of the closest match for that sequence. CW: colonized wood, KC: killed control, AC: abiotic control, EX: experimental treatment.

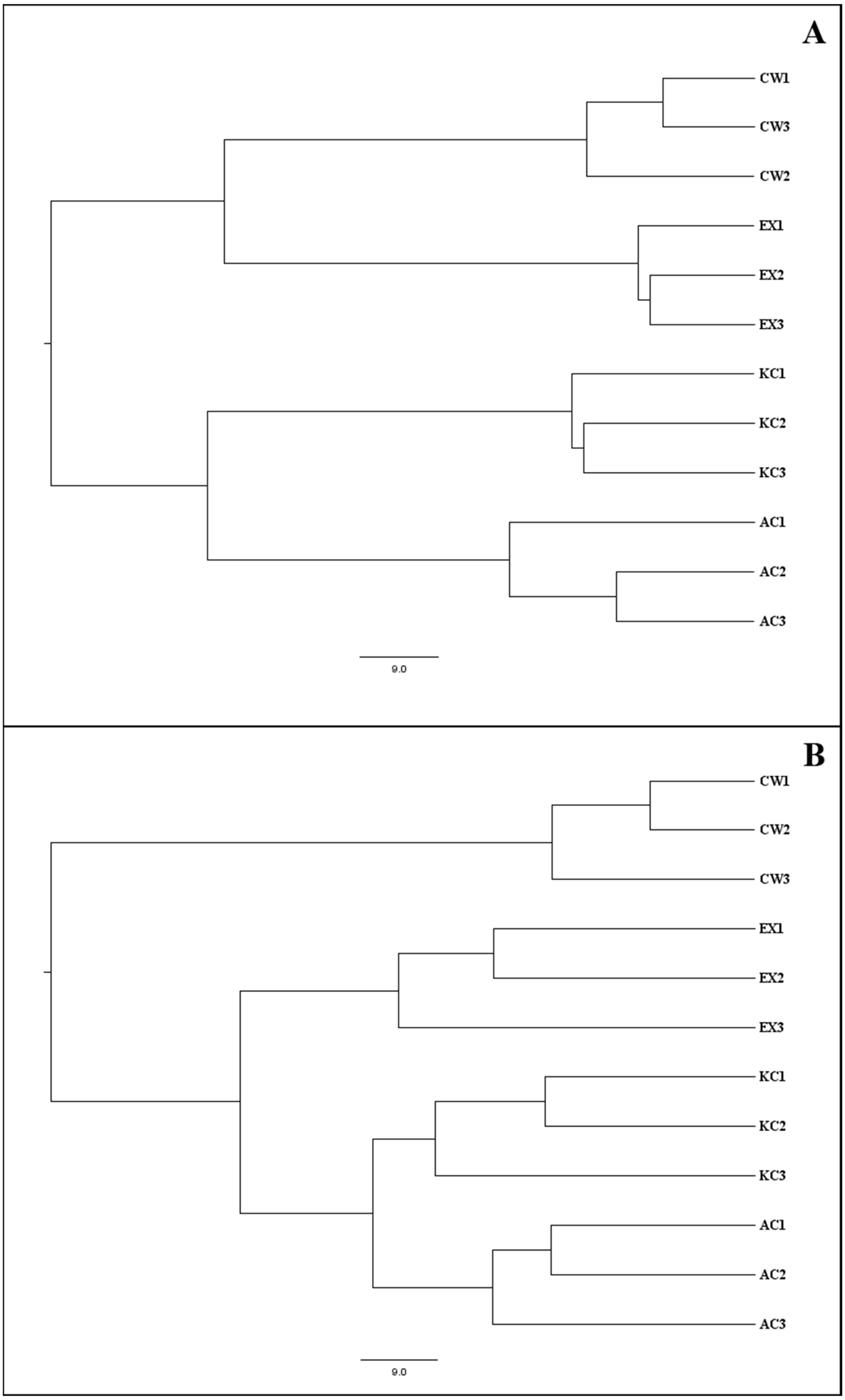

Figure S3. Dendrograms for fungal (A) and bacterial (B) communities of the different treatments, clustered with UPGMA from the DGGE similarity matrix calculated with Dice index. CW: colonized wood, KC: killed control, C: abiotic control, EX: experimental treatment.

Table S6. Phylogenetic affiliations of fungal ITS sequences obtained from the reactors after DGGE.

Band	Closest Blast[a] match	Accession number	Identity (%)	Phylogenetic affiliation (phylum, class, order)
1	*Ganoderma sessile*	MG654319.1	97.25	*Basidiomycota, Agaricomycetes, Polyporales*
2	*Ganoderma sessile*	MG654319.1	97.98	*Basidiomycota, Agaricomycetes, Polyporales*
3	*Ganoderma sessile*	MG654319.1	99.6	*Basidiomycota, Agaricomycetes, Polyporales*
4	*Ganoderma sessile*	MG654319.1	96.14	*Basidiomycota, Agaricomycetes, Polyporales*
5	*Ganoderma sessile*	MG654318.1	100	*Basidiomycota, Agaricomycetes, Polyporales*
6	*Ganoderma sessile*	MG654318.1	100	*Basidiomycota, Agaricomycetes, Polyporales*
7	*Ganoderma sessile*	MG654319.1	97.03	*Basidiomycota, Agaricomycetes, Polyporales*
8	*Ganoderma sessile*	MG654319.1	94.66	*Basidiomycota, Agaricomycetes, Polyporales*
9	*Ganoderma sessile*	MG654319.1	98.09	*Basidiomycota, Agaricomycetes, Polyporales*
10	*Ganoderma sessile*	MG654319.1	100	*Basidiomycota, Agaricomycetes, Polyporales*
11	*Ganoderma sessile*	MG654319.1	99.17	*Basidiomycota, Agaricomycetes, Polyporales*
12	*Ganoderma sessile*	MG654319.1	100	*Basidiomycota, Agaricomycetes, Polyporales*
13	*Ganoderma sessile*	MG654318.1	98.01	*Basidiomycota, Agaricomycetes, Polyporales*
14	*Ganoderma sessile*	MG654319.1	99.59	*Basidiomycota, Agaricomycetes, Polyporales*
15	*Ganoderma sessile*	MG654318.1	96.47	*Basidiomycota, Agaricomycetes, Polyporales*
16	*Chaetomium sp.*	KY132109.1	98.43	*Ascomycota, Sordariomycetes, Sordariales*
17	*Chaetomium sp.*	KY132109.1	99.03	*Ascomycota, Sordariomycetes, Sordariales*
18	*Chaetomium sp.*	KY132109.1	99.51	*Ascomycota, Sordariomycetes, Sordariales*
19	*Chaetomium sp.*	KY132109.1	99.01	*Ascomycota, Sordariomycetes, Sordariales*
20	*Chaetomium sp.*	KY132109.1	99.51	*Ascomycota, Sordariomycetes, Sordariales*
21	*Chaetomium sp.*	KY132109.1	99.51	*Ascomycota, Sordariomycetes, Sordariales*
22	uncultured *Apiotrichum*	OY844229.1	100	*Basidiomycota, Tremellomycetes, Trichosporonales*
23	uncultured *Apiotrichum*	OY844229.1	100	*Basidiomycota, Tremellomycetes, Trichosporonales*
24	uncultured *Apiotrichum*	OY844229.1	100	*Basidiomycota, Tremellomycetes, Trichosporonales*
25	*[Candida] sp. (uncertain placement)*	MT852063.1	99.59	*Ascomycota, Saccharomycetes, Saccharomycetales*
26	*[Candida] sp. (uncertain placement)*	MT852063.1	100	*Ascomycota, Saccharomycetes, Saccharomycetales*

Table S6. cont

Band	Closest Blast[a] match	Accession number	Identity (%)	Phylogenetic affiliation (phylum, class, order)
27	*[Candida] sp. (uncertain placement)*	MT852063.1	100	*Ascomycota, Saccharomycetes, Saccharomycetales*
28	*uncultured Apiotrichum*	OY844229.1	100	*Basidiomycota, Tremellomycetes, Trichosporonales*
29	*uncultured Apiotrichum*	OY844229.1	99.37	*Basidiomycota, Tremellomycetes, Trichosporonales*
30	*Naganishia sp.*	MK536807.1	98	*Basidiomycota, Tremellomycetes, Filobasidiales*
31	*Naganishia sp.*	MK536807.1	95.16	*Basidiomycota, Tremellomycetes, Filobasidiales*
32	*Naganishia sp.*	MK536807.1	98.03	*Basidiomycota, Tremellomycetes, Filobasidiales*
33	*Rhodotorula kratochvilovae*	LC390313.1	100	*Basidiomycota, Microbotryomycetes, Sporidiobolales*
34	*Rhodotorula kratochvilovae*	LC390313.1	98.34	*Basidiomycota, Microbotryomycetes, Sporidiobolales*
35	*Rhodotorula kratochvilovae*	LC390313.1	100	*Basidiomycota, Microbotryomycetes, Sporidiobolales*
36	*[Rhodotorula] nothofagi*	MT852233.1	100	*Basidiomycota, Microbotryomycetes, Sporidiobolales*
37	*[Rhodotorula] nothofagi*	MT852233.1	98.95	*Basidiomycota, Microbotryomycetes, Sporidiobolales*
38	*[Rhodotorula] nothofagi*	MT852233.1	99.47	*Basidiomycota, Microbotryomycetes, Sporidiobolales*
39	*Ascomycota sp.*	MH430776.1	100	*Ascomycota*
40	*Ascomycota sp.*	MH430776.1	98.1	*Ascomycota*
41	*Ascomycota sp.*	MH430776.1	97.67	*Ascomycota*
42	*Rhodotorula kratochvilovae*	LC390313.1	98.43	*Basidiomycota, Microbotryomycetes, Sporidiobolales*
43	*Rhodotorula kratochvilovae*	LC390313.1	100	*Basidiomycota, Microbotryomycetes, Sporidiobolales*
44	*Rhodotorula kratochvilovae*	LC390313.1	100	*Basidiomycota, Microbotryomycetes, Sporidiobolales*
45	*Rhodotorula kratochvilovae*	LC390313.1	100	*Basidiomycota, Microbotryomycetes, Sporidiobolales*
46	*Rhodotorula kratochvilovae*	LC390313.1	98.4	*Basidiomycota, Microbotryomycetes, Sporidiobolales*
47	*Rhodotorula kratochvilovae*	LC390313.1	100	*Basidiomycota, Microbotryomycetes, Sporidiobolales*
48	*[Rhodotorula] nothofagi*	MT852233.1	98.94	*Basidiomycota, Microbotryomycetes, Sporidiobolales*
49	*[Rhodotorula] nothofagi*	MT852233.1	98.94	*Basidiomycota, Microbotryomycetes, Sporidiobolales*
50	*[Rhodotorula] nothofagi*	MT852233.1	100	*Basidiomycota, Microbotryomycetes, Sporidiobolales*
51	*Penicillium brevicompactum*	MN577353.1	99.07	*Ascomycota, Eurotiomycetes, Eurotiales*
52	*Penicillium brevicompactum*	MN577353.1	100	*Ascomycota, Eurotiomycetes, Eurotiales*
53	*[Candida] sp. (uncertain placement)*	MT852063.1	100	*Ascomycota, Eurotiomycetes, Eurotiales*

Table S6. cont

Band	Closest Blast[a] match	Accession number	Identity (%)	Phylogenetic affiliation (phylum, class, order)
54	*Rhodotorula kratochvilovae*	LC390313.1	99.47	*Basidiomycota, Microbotryomycetes, Sporidiobolales*
55	*Rhodotorula kratochvilovae*	LC390313.1	97.93	*Basidiomycota, Microbotryomycetes, Sporidiobolales*
56	*Rhodotorula kratochvilovae*	LC390313.1	100	*Basidiomycota, Microbotryomycetes, Sporidiobolales*
57	*Rhodotorula kratochvilovae*	LC390313.1	100	*Basidiomycota, Microbotryomycetes, Sporidiobolales*
58	*Rhodotorula kratochvilovae*	LC390313.1	100	*Basidiomycota, Microbotryomycetes, Sporidiobolales*
59	*Cystobasidium slooffiae*	MK336491.1	97.74	*Basidiomycota, Cystobasidiomycetes, Cystobasidiales*
60	*Cystobasidium slooffiae*	MK336491.1	98.33	*Basidiomycota, Cystobasidiomycetes, Cystobasidiales*
61	*Ganoderma sessile*	MG654318.1	99.2	*Basidiomycota, Agaricomycetes, Polyporales*
62	*Ganoderma sessile*	MG654318.1	100	*Basidiomycota, Agaricomycetes, Polyporales*
63	*Ganoderma sessile*	MG654319.1	100	*Basidiomycota, Agaricomycetes, Polyporales*
64	*Ganoderma sessile*	MG654319.1	99.6	*Basidiomycota, Agaricomycetes, Polyporales*
65	*Ganoderma sessile*	MG654319.1	99.2	*Basidiomycota, Agaricomycetes, Polyporales*
66	*Ganoderma sessile*	MG654318.1	100	*Basidiomycota, Agaricomycetes, Polyporales*
67	*Cystobasidium slooffiae*	MK336491.1	100	*Basidiomycota, Cystobasidiomycetes, Cystobasidiales*
68	*Cystobasidium slooffiae*	MK336491.1	100	*Basidiomycota, Cystobasidiomycetes, Cystobasidiales*
69	*Cystobasidium slooffiae*	MK336491.1	100	*Basidiomycota, Cystobasidiomycetes, Cystobasidiales*
70	*Ganoderma sessile*	MG654319.1	100	*Basidiomycota, Agaricomycetes, Polyporales*
71	*Ganoderma sessile*	MG654319.1	97.22	*Basidiomycota, Agaricomycetes, Polyporales*
72	*Ganoderma sessile*	MG654319.1	96.47	*Basidiomycota, Agaricomycetes, Polyporales*
73	*Ganoderma sessile*	MG654319.1	98.41	*Basidiomycota, Agaricomycetes, Polyporales*
74	*Ganoderma sessile*	MG654319.1	99.6	*Basidiomycota, Agaricomycetes, Polyporales*
75	*Ganoderma sessile*	MG654318.1	97.61	*Basidiomycota, Agaricomycetes, Polyporales*
76	*Ganoderma sessile*	MG654319.1	99.13	*Basidiomycota, Agaricomycetes, Polyporales*
77	*Ganoderma sessile*	MG654318.1	100	*Basidiomycota, Agaricomycetes, Polyporales*
78	*Ganoderma sessile*	MG654319.1	98.78	*Basidiomycota, Agaricomycetes, Polyporales*
79	*Ganoderma sessile*	MG654319.1	97.98	*Basidiomycota, Agaricomycetes, Polyporales*
80	*Ganoderma sessile*	LC390313.1	98.39	*Basidiomycota, Agaricomycetes, Polyporales*

Table S6. cont

Band	Closest Blast[a] match	Accession number	Identity (%)	Phylogenetic affiliation (phylum, class, order)
81	*Ganoderma sessile*	MG654319.1	99.2	*Basidiomycota, Agaricomycetes, Polyporales*
82	*Ganoderma sessile*	MG654318.1	100	*Basidiomycota, Agaricomycetes, Polyporales*
83	*Ganoderma sessile*	MG654318.1	98.01	*Basidiomycota, Agaricomycetes, Polyporales*
84	*Ganoderma sessile*	MG654318.1	98.4	*Basidiomycota, Agaricomycetes, Polyporales*
85	uncultured *Apiotrichum*	OY844229.1	98.12	*Basidiomycota, Tremellomycetes, Trichosporonales*
86	uncultured *Apiotrichum*	OY844229.1	98.03	*Basidiomycota, Tremellomycetes, Trichosporonales*
87	uncultured *Apiotrichum*	OY844229.1	99.35	*Basidiomycota, Tremellomycetes, Trichosporonales*

a: BLAST (Basic local alignment search tool) (Camacho et al., 2009).

Table S7. Phylogenetic affiliations of bacterial 16S rRNA gene sequences obtained from the reactors after DGGE.

Band	Closest Blast[a] match	Accession number	Identity %	Phylogenetic affiliation (phylum, class, order)
1	*Achromobacter xylosoxidans*	JQ746447.1	96.33	*Proteobacteria, Betaproteobacteria, Burkholderiales*
2	*Achromobacter sp.*	MH130293.1	99.02	*Proteobacteria, Betaproteobacteria, Burkholderiales*
3	*Achromobacter marplatensis*	MT322272.1	98.24	*Proteobacteria, Betaproteobacteria, Burkholderiales*
4	*Achromobacter sp.*	OL851711.1	98.42	*Proteobacteria, Betaproteobacteria, Burkholderiales*
5	*Achromobacter sp.*	MF289213.1	99.02	*Proteobacteria, Betaproteobacteria, Burkholderiales*
6	*Achromobacter sp.*	MF289213.1	100	*Proteobacteria, Betaproteobacteria, Burkholderiales*
7	*Burkholderiaceae bacterium*	MK463013.1	98.8	*Proteobacteria, Betaproteobacteria, Burkholderiales*
8	*Burkholderia sp.*	MK373524.1	98.84	*Proteobacteria, Betaproteobacteria, Burkholderiales*
9	uncultured *Burkholderiaceae bacterium*	MT002711.1	97.06	*Proteobacteria, Betaproteobacteria, Burkholderiales*
10	*Rhodococcus sp.*	ON422169.1	99.01	*Actinobacteria, Actinomycetia, Mycobacteriales*
11	*Rhodococcus sp.*	ON422169.1	99.6	*Actinobacteria, Actinomycetia, Mycobacteriales*
12	*Rhodococcus sp.*	ON422169.1	99.6	*Actinobacteria, Actinomycetia, Mycobacteriales*
13	*Terriglobus sp.*	KX555422.1	98.52	*Acidobacteria, Terriglobia, Terriglobales*
14	*Terriglobus sp.*	KX555422.1	96.36	*Acidobacteria, Terriglobia, Terriglobales*
15	*Rhizobium sp. rob18*	AJ271901.1	96.49	*Proteobacteria, Alphaproteobacteria, Hyphomicrobiales*
16	*Rhizobium sp. rob18*	AJ271901.1	99.18	*Proteobacteria, Alphaproteobacteria, Hyphomicrobiales*
17	*Terriglobus sp.*	KX555422.1	99.98	*Acidobacteria, Terriglobia, Terriglobales*
18	*Acidisoma sp.*	KY907904.1	96.96	*Proteobacteria, Alphaproteobacteria, Rhodospirillales*
19	*Luteibacter rhizovicinus*	OQ346266.1	100	*Proteobacteria, Gammaproteobacteria, Lysobacterales*
20	uncultured *Sphingobacteriales bacterium*	FJ475373.1	96.95	*Bacterioidetes, Sphingobacteriia, Sphingobacteriales*
21	uncultured *Sphingobacteriales bacterium*	FJ475373.1	95.49	*Bacterioidetes, Sphingobacteriia, Sphingobacteriales*
22	*Rhizobium sp. rob18*	AJ271901.1	99.79	*Proteobacteria, Alphaproteobacteria, Hyphomicrobiales*
23	*Rhizobium sp. rob18*	AJ271901.1	99.79	*Proteobacteria, Alphaproteobacteria, Hyphomicrobiales*
24	*Terriglobus sp.*	KX555422.1	94.68	*Acidobacteria, Terriglobia, Terriglobales*
25	*Terriglobus sp.*	KX555422.1	99.59	*Acidobacteria, Terriglobia, Terriglobales*
26	*Acidisoma sp.*	KY907904.1	94.32	*Proteobacteria, Alphaproteobacteria, Rhodospirillales*

Table S7. cont

Band	Closest Blast[a] match	Accession number	Identity %	Phylogenetic affiliation (phylum, class, order)
27	*Luteibacter sp. AK20-111*	KP899142.1	99.81	*Proteobacteria, Gammaproteobacteria, Lysobacterales*
28	uncultured *Sphingobacteriia bacterium*	FJ475373.1	94.82	*Bacterioidetes, Sphingobacteriia, Sphingobacteriales*
29	*Terriglobus sp.*	KX555422.1	96.13	*Acidobacteria, Terriglobia, Terriglobales*
30	*Rhizobium sp.*	KY117505.1	98.65	*Proteobacteria, Alphaproteobacteria, Hyphomicrobiales*
31	*Terriglobus sp.*	KX555422.1	95.97	*Acidobacteria, Terriglobia, Terriglobales*
32	*Terriglobus sp.*	KX555422.1	96.45	*Acidobacteria, Terriglobia, Terriglobales*
33	*Luteibacter rhizovicinus*	MT281377.1	100	*Proteobacteria, Gammaproteobacteria, Lysobacterales*
34	*Achromobacter sp.*	MH130292.1	100	*Proteobacteria, Betaproteobacteria, Burkholderiales*
35	*Terriglobus sp.*	KX555422.1	98.98	*Acidobacteria, Terriglobia, Terriglobales*
36	*Terriglobus sp.*	KX555422.1	99.36	*Acidobacteria, Terriglobia, Terriglobales*
37	*Terriglobus sp.*	KX555422.1	97.13	*Acidobacteria, Terriglobia, Terriglobales*
38	*Novosphingobium sp. Y3-5*	KT452772.1	98.26	*Proteobacteria, Alphaproteobacteria, Sphingomonadales*
39	*Luteibacter sp. AK20-111*	KP899142.1	98.73	*Proteobacteria, Gammaproteobacteria, Lysobacterales*
40	*Luteibacter rhizovicinus*	MT281377.1	97.47	*Proteobacteria, Gammaproteobacteria, Lysobacterales*
41	*Acidisoma sp.*	KY907904.1	99.17	*Proteobacteria, Alphaproteobacteria, Rhodospirillales*
42	*Acidisoma sp.*	KY907904.1	98.98	*Proteobacteria, Alphaproteobacteria, Rhodospirillales*
43	*Terriglobus sp.*	KX555422.1	99.39	*Acidobacteria, Terriglobia, Terriglobales*
44	*Terriglobus sp.*	KX555422.1	97.34	*Acidobacteria, Terriglobia, Terriglobales*
45	*Terriglobus sp.*	KX555422.1	97.22	*Acidobacteria, Terriglobia, Terriglobales*
46	*Granulicella sp.*	MN193335.1	98.78	*Acidobacteria, Terriglobia, Terriglobales*
47	*Granulicella sp.*	MN193335.1	97.99	*Acidobacteria, Terriglobia, Terriglobales*
48	*Terriglobus sp.*	KX555422.1	95.06	*Acidobacteria, Terriglobia, Terriglobales*
49	*Novosphingobium sp. Y3-5*	KT452772.1	99.18	*Proteobacteria, Alphaproteobacteria, Sphingomonadales*
50	*Novosphingobium sp. Y3-5*	KT452772.1	99.38	*Proteobacteria, Alphaproteobacteria, Sphingomonadales*
51	*Curtobacterium sp.*	MK618609.1	96.94	*Actinobacteria, Actinomycetia, Micrococcales*
52	*Curtobacterium sp.*	MK618609.1	98.98	*Actinobacteria, Actinomycetia, Micrococcales*
53	*Dyella sp.*	KP899142.1	98.74	*Proteobacteria, Gammaproteobacteria, Lysobacterales*

Table S7. cont

Band	Closest Blast[a] match	Accession number	Identity %	Phylogenetic affiliation (phylum, class, order)
54	*Acidisoma sp.*	OR327596.1	95.28	*Proteobacteria, Alphaproteobacteria, Rhodospirillales*
55	*Luteibacter sp.*	MT012247.1	96.39	*Proteobacteria, Gammaproteobacteria, Lysobacterales*
56	*Luteibacter sp.*	MT012247.1	98.2	*Proteobacteria, Gammaproteobacteria, Lysobacterales*
57	*Terriglobus sp.*	KX555422.1	99.38	*Acidobacteria, Terriglobia, Terriglobales*
58	*Terriglobus sp.*	KX555422.1	99.39	*Acidobacteria, Terriglobia, Terriglobales*
59	*Terriglobus sp.*	KX555422.1	99.39	*Acidobacteria, Terriglobia, Terriglobales*
60	*Terriglobus sp.*	KX555422.1	99.17	*Acidobacteria, Terriglobia, Terriglobales*
61	*Terriglobus sp.*	KX555422.1	98.79	*Acidobacteria, Terriglobia, Terriglobales*
62	*Terriglobus sp.*	KX555422.1	95.16	*Acidobacteria, Terriglobia, Terriglobales*
63	*Terriglobus sp.*	KX555422.1	97.89	*Acidobacteria, Terriglobia, Terriglobales*
64	*Terriglobus sp.*	KX555422.1	97.67	*Acidobacteria, Terriglobia, Terriglobales*
65	*Terriglobus sp.*	KX555422.1	98.73	*Acidobacteria, Terriglobia, Terriglobales*
66	*Rhizobium sp. rob18*	AJ271901.1	98.57	*Proteobacteria, Alphaproteobacteria, Hyphomicrobiales*
67	*Rhizobium sp. rob18*	AJ271901.1	95.75	*Proteobacteria, Alphaproteobacteria, Hyphomicrobiales*
68	*Caballeronia sordidicola*	OR373319.1	95.17	*Proteobacteria, Betaproteobacteria, Burkholderiales*
69	*Caballeronia sp.*	OR373319.1	99.22	*Proteobacteria, Betaproteobacteria, Burkholderiales*
70	*Caballeronia sp.*	OR373319.1	97.68	*Proteobacteria, Betaproteobacteria, Burkholderiales*
71	*Terriglobus sp.*	KX555422.1	98.36	*Acidobacteria, Terriglobia, Terriglobales*
72	*Terriglobus roseus*	LT629690.1	99.39	*Acidobacteria, Terriglobia, Terriglobales*
73	*Terriglobus roseus*	LT629690.1	99.19	*Acidobacteria, Terriglobia, Terriglobales*

a: BLAST (Basic local alignment search tool) (Camacho et al., 2009).

Table S8. Standard curve results for the qPCRs performed in this study

qPCR target	Efficiency	R^2	Calibrated curve
Ganoderma sp.	101.27%	0.990	Ct=-3.292*log(conc)+20.673
Total fungi	96.31%	0.998	Ct=-3.414*log(conc)+34.853
Total bacteria	100.03%	0.997	Ct=-3.321*log(conc)+30.359

References

1. Camacho, C.; Coulouris, G.; Avagyan, V.; Ma, N.; Papadopoulos, J.; Bealer, K.; Madden, T.L. BLAST+: Architecture and Applications. *BMC Bioinformatics* **2009**, *10*, 421. https://doi.org/10.1186/1471-2105-10-421.
2. Dorn-In, S.; Bassitta, R.; Schwaiger, K.; Bauer, J.; Hölzel, C.S. Specific Amplification of Bacterial DNA by Optimized So-Called Universal Bacterial Primers in Samples Rich of Plant DNA. *J Microbiol Methods* **2015**, *113*, 50–56. https://doi.org/10.1016/J.MIMET.2015.04.001.
3. Gardes, M.; Bruns, T.D. ITS Primers with Enhanced Specificity for Basidiomycetes - Application to the Identification of Mycorrhizae and Rusts. *Mol Ecol* **1993**, *2*, 113–118. https://doi.org/10.1111/J.1365-294X.1993.TB00005.X.
4. Guglielmo, F.; Bergemann, S.E.; Gonthier, P.; Nicolotti, G.; Garbelotto, M. A Multiplex PCR-based Method for the Detection and Early Identification of Wood Rotting Fungi in Standing Trees. *J Appl Microbiol* **2007**, *103*, 1490–1507. https://doi.org/doi:10.1111/J.1365-2672.2007.03378.X.
5. Maza-Márquez, P.; Vílchez-Vargas, R.; González-Martínez, A.; González-López, J.; Rodelas, B. Assessing the Abundance of Fungal Populations in a Full-Scale Membrane Bioreactor (MBR) Treating Urban Wastewater by Using Quantitative PCR (QPCR). *J Environ Manage* **2018**, *223*, 1–8. https://doi.org/doi:10.1016/J.JENVMAN.2018.05.093.
6. Muyzer, G.; Hottenträger, S.; Teske, A.; Wawer, C. Denaturing Gradient Gel Electrophoresis of PCR-Amplified 16S RDNA - a New Molecular Approach to Analyze the Genetic Diversity of Mixed Microbial Communities. In *Molecular Microbial Ecology Manual*; Akkermans, A.D.L., van Elsas, J.D., de Bruijn, F.J., Eds.; Kluwer Academic Publishers: Dordretch, The Netherlands, 1996; pp. 1–23.
7. Muyzer, G.; De Waal, E.C.; Uitterlinden, A.G. Profiling of Complex Microbial Populations by Denaturing Gradient Gel Electrophoresis Analysis of Polymerase Chain Reaction-Amplified Genes Coding for 16S RRNA. *Appl Environ Microbiol* **1993**, *59*, 695. https://doi.org/10.1128/AEM.59.3.695-700.1993.
8. White, T.J.; Bruns, T.; Lee, S.; Taylor, J. Amplification and Direct Sequencing of Fungal Ribosomal RNA Genes for Phylogenetics. In *PCR protocols: a guide to methods and applications* ; Michael, A.I., Gelfand, D.H., Sninsky, J.J., White, T.J., Eds.; Academic Press: Cambridge, MA, USA, 1990; pp. 315–322 ISBN 9780080886718.
